# Mouse models of human TB pathology: roles in the analysis of necrosis and the development of host-directed therapies

**DOI:** 10.1007/s00281-015-0538-9

**Published:** 2015-11-05

**Authors:** Igor Kramnik, Gillian Beamer

**Affiliations:** Pulmonary Center, Department of Medicine, National Emerging Infectious Diseases Laboratories, Boston University School of Medicine, 620 Albany Street, Room 501, Boston, MA 02118 USA; Department of Infectious Disease and Global Health, Cummings School of Veterinary Medicine, Tufts University, 200 Westboro Rd, Bldg 20, Grafton, MA 01536 USA

**Keywords:** Tuberculosis, Granuloma, Necrosis, Animal models, Inbred mice, Mechanisms, Host-directed therapies, *sst1*

## Abstract

A key aspect of TB pathogenesis that maintains *Mycobacterium tuberculosis* in the human population is the ability to cause necrosis in pulmonary lesions. As co-evolution shaped *M. tuberculosis* (*M.tb*) and human responses, the complete TB disease profile and lesion manifestation are not fully reproduced by any animal model. However, animal models are absolutely critical to understand how infection with virulent *M.tb* generates outcomes necessary for the pathogen transmission and evolutionary success. In humans, a wide spectrum of TB outcomes has been recognized based on clinical and epidemiological data. In mice, there is clear genetic basis for susceptibility. Although the spectra of human and mouse TB do not completely overlap, comparison of human TB with mouse lesions across genetically diverse strains firmly establishes points of convergence. By embracing the genetic heterogeneity of the mouse population, we gain tremendous advantage in the quest for suitable in vivo models. Below, we review genetically defined mouse models that recapitulate a key element of *M.tb* pathogenesis—induction of necrotic TB lesions in the lungs—and discuss how these models may reflect TB stratification and pathogenesis in humans. The approach ensures that roles that mouse models play in basic and translational TB research will continue to increase allowing researchers to address fundamental questions of TB pathogenesis and bacterial physiology in vivo using this well-defined, reproducible, and cost-efficient system. Combination of the new generation mouse models with advanced imaging technologies will also allow rapid and inexpensive assessment of experimental vaccines and therapies prior to testing in larger animals and clinical trials.

## Pulmonary necrosis in *M.tb* life cycle

A long history of human co-evolution with *Mycobacterium tuberculosis* (*M.tb*) suggests that unique immune mechanisms have evolved explaining substantial resistance of modern humans to the disease [1–3]. However, our species also proved to be an ideal host for *M.tb*, such that the bacteria lost the need for any other environmental niche and relied entirely on modifying human body for every stage of its life cycle. It is generally accepted that to establish new infection, *M.tb* reaches terminal airways in small aerosol particles generated during cough. Those aerosols are generated from lung cavities where *M.tb* accumulates in large quantities, perhaps in biofilms, at the air interface, effectively sequestered from host immunity. Thus, from the evolutionary standpoint, *M.tb* is an obligate lung pathogen, such that substantial *M.tb*-induced destruction of lung tissue is absolutely required for direct transmission to other humans. Although the bacteria are capable of achieving this goal only in a relatively small fraction of the infected hosts, this is sufficient for stable colonization of its unique natural habitat, *Homo sapiens.* Therefore, understanding mechanisms of host susceptibility enabling *M.tb*, transmission is necessary to counter its evolutionary-refined virulence strategy most effectively.

Although animal models have been a lynchpin for mechanistic studies of TB since Robert Koch’s discovery of the pathogen, little progress has been made in understanding mechanisms of pulmonary destruction caused by *M.tb*. Recent experimental work has demonstrated that despite the uniqueness of human species as a host to *M.tb*, mice could be reliably used to study mechanisms of necrotization in TB granulomas and formation of lung cavities. Thus, the powerful arsenal of mouse genetic methods can be utilized to mechanistically dissect those crucial transitions during the natural course of TB infection. Below, we review genetically defined mouse models that recapitulate necrotic TB lesions in the lungs and discuss how these models may reflect TB pathogenesis in humans.

### Necrosis occurs at various stages of TB infection

There are at least two distinct stages of *M.tb* infection at which necrosis can occur: (1) at the initial stage of lung colonization, which leads to necrosis of individual or small clusters of macrophages in a primary granuloma, and (2) during advanced disease where large areas of the lung are effaced by coalescing granulomas and tuberculous pneumonia. Thus, necrosis delimits the life cycle of *M.tb* within an individual and plays an important role in both establishing persistent infection initially as well as in the final exit and transmission to a new host. There are many important mechanistic differences between the two stages.

The initial contact of *M.tb* with the host occurs in highly aerated environment, presumably with alveolar macrophages in a context of normal lung tissue. The alveolar macrophages are permissive to *M.tb*, which establishes its first replicative niche in this cell. Subsequent interactions with lung epithelium and possibly innate T cells lead to recruitment of inflammatory cells from circulation and establishing clusters of myeloid cells that contain the bacteria. These early dynamics cannot be addressed directly in natural lung environment in humans, but the zebrafish model of infection with *Mycobacterium marinum* provides a detailed view of cellular recruitment and interactions that establish nascent microgranulomas [4–6] before antigen-specific immunity develops. Although zebrafish do not have lungs, this model allows detailed cell trafficking studies in vivo and elegantly shows that macrophage death spreads bacilli to adjacent recruited macrophages within the same granuloma. Furthermore, when the recruitment of myeloid cells fails to contain *M.tb*, the bacilli replicate extracellularly with conspicuous formation of cords [7]. Multiple host responses contribute to macrophage necrosis, including alterations in lipid mediators and increased TNFα production or deficient recruitment of new myeloid cells to the site of infection [8]. Similarly, in the lungs of *M.tb*-infected C3HeB/FeJ mice (abbreviated HeB), early micronecrotic lesions form 2–3 weeks post-infection, when spread of *M.tb* to adjacent inflammatory cells, as well as robust extracellular replication are observed [9]. In both the zebrafish model and HeB mice, the microgranulomas undergoing necrosis are composed primarily of myeloid cells (macrophages and some granulocytes). In contrast, different host-pathogen dynamics are observed in the relatively resistant mouse strain C57BL/6 (B6), where lesions are non-necrotic, contain few neutrophils, and bacilli remain intracellular.

The early granulomas are composed primarily of myeloid cells that initially act autonomously to restrict the bacterial growth and spread. However, adaptive T cell-mediated immunity is necessary to contain further progression and necrotization of granulomas. The bacterial spread is unstoppable in T cell -deficient mice, where mycobacteria replicate in unrestricted manner and destroy the infected tissue. Large areas of necrotic inflammation, massive bacterial loads, and extensive neutrophil infiltration are typical for this type of progression, which, however, lacks the characteristics of organized granulomas. Thus, the early TB granulomas can follow necrotic and non-necrotic trajectories depending on the myeloid cell intrinsic capacity and help of *M.tb*-induced T lymphocytes producing IFNγ (Th1-type response). In both cases, however, they serve to constrain *M.tb* and prevent dissemination. In the case of more efficient immune response in resistant hosts, primary granulomas may be sterilized over time and undergo calcification. In permissive but immune-competent hosts, however, small necrotic granulomas establish a nidus of persistent infection, which can later reactivate and cause post-primary TB.

The exit and transmission strategy of *M.tb* at the end of its life cycle is entirely dependent on granuloma spread and necrosis leading to formation of lung cavities. Those necrotic lesions become *M.tb* sanctuaries sequestering the pathogen from the host immune system and allowing its replication and transmission via aerosols. This transition occurs in immune-competent hosts that successfully controlled the primary infection. Thus, the local mechanisms and the dynamics of necrosis at the advanced disease stage are not the same as in primary lesions and the organ- and organism-scale factors may play bigger or different roles. Two different models of necrosis in advanced TB have been proposed:

Model 1: This is the gradual necrotization and local expansion of organized granulomas, including formation and coalescense of satellite granulomas. Accumulation of dead macrophages that fail to survive intracellular *M.tb* infection is a primary source of the caseous necrotic masses.
Model 2: This is the rapid dissemination of *M.tb* from chronic lesion causing tuberculous pneumonia, where necrosis formation may be associated with thrombosed blood vessels and infarcted regions of the lungs [10, 11]. Subsequently, *M.tb* bacilli, dead inflammatory cells, and dead lung tissue may be sequestered in a fibrous capsule to re-contain the pathogen.

Clearly mechanisms and consequences of lung necrosis need to be considered within the genetic and immunological context of the host and the stage of disease. We also would like to emphasize the distinction between systemic, lung, and cellular levels that contribute to necrosis and will discuss hypotheses related to necrosis in that order.

### Systemic host factors that contribute to necrosis

Arnold Rich pointed out that “tubercule bacilli have very little power of producing necrosis of tissue in the normal (non-hypersensitive) body… Injected locally in large amount it does not kill tissue… A fraction of the number of bacilli, which could be injected into the tissues without causing necrosis in the normal body, produces violent inflammation and extensive necrosis in the hypersensitive one”([12], p. 350). The dual role of inflammation was further investigated by Arthur Dannenberg, who distinguished cytotoxic delayed-type hypersensitivity and macrophage-activating cell-mediated immunity and pointed to a therapeutic potential of manipulating their balance. He used the rabbit model to experimentally reproduce those types [13] and demonstrated that both types of responses were driven by systemic immunity. However, at that stage, it was impossible to identify molecular determinants of “protective” versus “pathogenic” inflammation as specific therapeutic targets. Those classical experiments demonstrating transition from mild protective to “violent” destructive inflammation implicated systemic host immune response to *M.tb* in evolution of the host protective granulomas into pathogen-favoring ones. However, specific mechanisms of adaptive immunity causing necrosis in TB granulomas still remain hypothetical [14].

### Local granuloma factors that contribute to necrosis

Mechanistically, the formation of necrotic TB lesions and liquefaction of cellular debris were attributed to release of hydrolytic enzymes by macrophages and neutrophils. Among them are MMP1 and MMP8 [15–18] and serine proteases [19], which degrade extracellular matrix proteins and basement membranes, participating in lung tissue destruction and stimulating fibrosis. Although those enzymes may be attractive therapeutic targets, they are likely to be executors but not the root causes of lung tissue necrosis.

Recent studies demonstrate that dysbalance of inflammatory pathways may lead to necrotizing inflammation. For example, activation of type I interferon (IFN-I) pathway by instilling tlr3 ligand poly(I:C) in the lungs promoted the development of acute necrotic TB lesions via excessive recruitment of myeloid cells [20]. In another model, overexpression of IL-13 using T cell-specific promoter generated conditions for the development of well-organized necrotic lung granulomas [21]. Interestingly, the IL-13 overexpression did not reduce Th1 responses, which would explain the necrotization by suppression of the essential host resistance pathway. Alternatively, increased sensitivity of alternatively activated macrophages to cytotoxic activity of TNFα in granulomas may be involved, as hypothesized previously by Rook and coauthors [22]. Another model of necrotic TB lesions has been generated using temporal inactivation of essential mechanisms of resistance, such as administration of NO inhibitor and neutralization of IFNγ using injections of monoclonal antibodies in mice infected with *M.tb* intradermally [19].

An important concept emerged recently based on studies of heterogeneous TB granulomas in non-human primates (NHP) using combination of live imaging, analysis of RNA expression patterns, *M.tb* loads, histopathology, and computational modeling [23–25]. The iNOS and arginase-1 protein expression in granulomas in situ were used as surrogate markers for the M1 and M2 macrophage phenotypes, respectively. The M1/M2 balance emerged as a best correlate of the granuloma outcome. Double-positive cells were found in granuloma walls, and phenotypically M1 cells increased towards the necrotic center. In this study, the higher M2 activation did not correlate with suppression of Th1 responses, as well. In a mouse model, the expression of arginase-1 was shown to be modestly protective in granulomas in the absence of iNOS, and simultaneous Arg1 and iNOS inactivation resulted in exuberant necrotic inflammation [26]. Both of those observations are more consistent with a possibility that the arginase-1 pathway does not suppress the NO-mediated effector pathway, but Arg1 is activated as a backup effector mechanism, when the more efficient NO-mediated effector pathway fails to control the bacteria, for example due to hypoxia in large progressive lesions. Taken together, the NHP provided a unique data set unattainable in the mouse model, meanwhile the mouse model provided a novel mechanistic insight for interpreting the NHP data.

### Factors that contribute to macrophage necrosis

At a cellular level, mechanisms of macrophage death directly caused by *M.tb* are under intense investigation. Roughly, they can be divided into two categories: (1) active mechanisms, whereby virulent mycobacteria or host produce toxic molecular mediators that cause macrophage death, and (2) passive mechanisms, where bacillary replication in macrophages results in lysis, essentially a “load-driven” death. The former category focuses on specific pathways that determine macrophage death modality. For example, the balance of lipid mediators PgE2 and lipoxin A4 has been shown to control membrane repair of the infected macrophages and apoptotic vs necrotic cell death [27, 28], as well as production of TNFα [29], whereas the higher concentrations of TNFα induced necroptotic pathway in macrophages [30]. The necrotic macrophage death is perceived as more detrimental for the host that might be associated with necrotic granuloma formation in vivo, while apoptotic death has been associated with host resistance and bacterial control [27, 31, 32].

*M.tb* produces multiple products that induce macrophage necrosis in vitro. Cord factor (trehalose 6,6′-dimycolate or TDM) is a mycolic acid-rich membrane glycolipid identified decades ago and is a well-known virulence and immunomodulatory factor of *M.tb.* TDM is particularly interesting because it may induce rapid macrophage necrosis. Early secreted antigenic target-6 (ESAT-6) is a virulence factor encoded within the RD1 region [33], which is absent from the vaccine strain *Mycobacterium bovis* BCG. ESAT-6 triggers necrosis directly [34] or indirectly by phagosomal rupture allowing *M.tb* access to the cytoplasm [35, 36] and activation of NLRP3 inflammasome [37]. Another cytotoxin CpnT has been recently discovered that kills macrophages via hydrolysing macrophage NAD [38, 39]. However, those toxic factors are not sufficient to induce necrotic granulomas in the genetically resistant mice. Meanwhile, even avirulent (ESAT-6 negative) *M. bovis* BCG was capable of inducing granuloma necrosis in immunodeficient Mendelian susceptibility to mycobacterial diseases (MSMD) human patients or T cell-deficient mice. The later facts are consistent with a burst size hypothesis, where unrestricted growth of intracellular mycobacteria results in achieving maximal load and eventually death of the infected cell [40]. A hybrid hypothesis stipulates that membrane-toxic mycobacterial products produced intracellularly may allow *M.tb* escape from vacuole to cytoplasm, where it can either replicate more rapidly to achieve the burst size or actively induce macrophage cell death via activation of type I IFN pathway [41, 42].

This brief review of literature suggests the co-existence, and potential cooperation, of multiple pathways leading to necrosis of granulomas. Here, we would like to emphasize that causality of necrosis may significantly differ between animal hosts and experimental systems, as well as between human patients.

## Genetic variation in mice controls necrotizing responses to *M.tb*

**Fig. 1:**
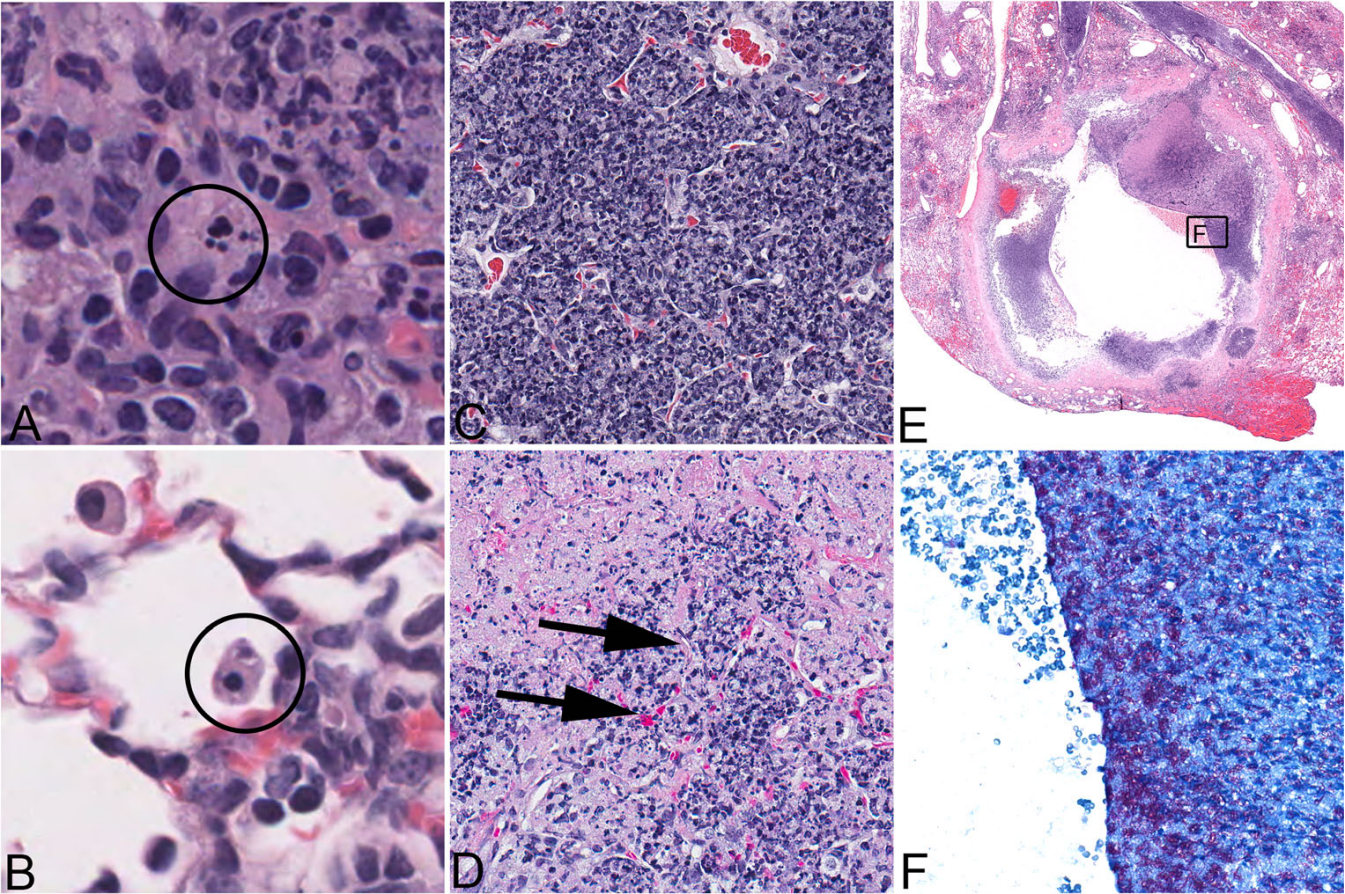
Manifestation of necrotizing responses and cavitation induced in vivo in the lungs of mice experimentally infected with *Mycobacterium tuberculosis*. Formalin-fixed, paraffin-embedded lung sections were stained with hematoxylin and eosin (**a** through **d**) or stained with a modification of Ziehl-Neelsen’s acid-fast method (**f**). Death can be observed in individual macrophages as apoptosis recognized by nuclear fragmentation, magnified 1,000 times, *encircled* (**a**). Necrosis of an individual macrophage in an alveolar space is recognized by cytoplasmic eosinophilia and a pyknotic, condensed nucleus, magnified 1,000 times (**b**). Alveolar septae are present but difficult to recognize due to accumulation of dead inflammatory cells and cellular debris within alveolar air spaces, magnified 400 times (**c**). As the necrotizing process continues, fibrin thrombosis of capillaries is observed as a transition from a capillary with red blood cells to a capillary containing eosinophilic fibrillar material consistent with fibrin (*arrows*), magnified 400 times (**d**). Finally, complete destruction of alveolar septae allows necrotic regions to coalesce and undergo liquefaction and removal of necrotic material, thus contributing to cavity formation, magnified 20 times normal (**e**). Cavities contain variable necrotic debris in which abundant acid-fast bacilli are detected, magnified 400 times (**f**). (**a**–**d**) necrotic lung lesions in supersusceptible DO mouse; (**e**–**f**) cavity in the lung of a CBA/J mouse

Many animal models are used to study *M.tb* lesions including mice, guinea pigs, rabbits, non-human primates, bovine calves, zebrafish, rats, ferrets, mini pigs, fruit flies, nematodes, planarians, and even amoebas [43–56]. Due to the small size, cost-efficiency, and availability of reagents, mice are widely used and have been crucial for determining immunological requirements that restrict *M.tb* growth [57, 58]. However, the two most commonly used inbred laboratory mouse strains (C57BL/6 and BALB/c) do not develop necrotizing lesions [56, 59, 60] which, unfortunately, have led to general criticism of the mouse model. When considering responses at the mouse population level, however, it becomes apparent that necrotizing responses to *M.tb* commonly occur in inbred (C3HeB/FeJ, DBA/2, CBA/J, I/St), inbred crossed (HET3), and even outbred crossed (Diversity Outbred) mice [61–67]. The spectrum of necrosis includes apoptosis and necrosis of individual macrophages, necrotizing granulomas with fibrotic capsule, tuberculous pneumonia with intra-alveolar neutrophilic exudates, and fibrin thrombosis of lung alveolar capillaries (Fig. 1a–d). Similar necrotizing responses are associated with and appear to precede cavity formation in humans and larger animal models (rabbits and NHPs) [68–70]. Although cavities were not reported in mice in the past, their formation has been well documented recently in C3HeB/FeJ [71, 72] and CBA/J mice (Fig. 1e–f) following their typical necrotizing responses.

Granulomas are often described with stereotypical organization: focal aggregates of central myeloid cells with peripheral lymphocytes and a fibrous capsule that separates the immune cells from the adjacent normal tissue. This is a simple model to envision; however, in reality, a significant heterogeneity between granulomas is observed even within the same individual in the clinic and in experimental animal models. Necrotic granulomas may develop necrotic centers (necrotizing granulomas) or be infiltrated by neutrophils (suppurative granulomas), although the conditions which drive those transitions are not fully known. Recent studies in C3HeB/FeJ mice link necrotizing structural and cellular responses to *M.tb* with morbidity [72, 73]. Type I, II, and III granulomas were characterized, but the lesions are not quite stereotypical granulomas mentioned above. Regardless of terminology, highest morbidity was observed in type II lesions, which were dominated by necrotizing responses: unencapsulated tuberculous pneumonia with macrophages, neutrophils, necrotic cell debris packed within alveolar spaces, and abundant intra- and extracellular bacilli. Type I lesions were smaller necrotizing lesions with central necrosis surrounded by macrophages, neutrophils, and peripheral fibrosis with abundant extracellular bacilli in the necrotic center and intracellular bacilli in foamy macrophages at the periphery. Type III were non-necrotic lesions composed of mononuclear cells and macrophages in alveoli, perivascular and peribronchiolar lymphocytic aggregates, and alveolar septal interstitial fibrosis. *M.tb* bacilli were intracellular within macrophages and relatively scarce. These lesion types are not unique to C3HeB/FeJ mice. We observed lesions identical to type I and type III in chronically infected CBA/J mice but not type II [64]. We also recently reported a similar spectrum in *M.tb-*infected Diversity Outbred mice. Highest morbidity occurred in the individuals that developed necrotizing neutrophilic tuberculous pneumonia (i.e., type II lesions) and these same mice had the highest bacillary burden [66]. We additionally observed capillary thrombosis in type II lesions [66] with loss of differential staining suggestive of infarction and coagulation necrosis. It is likely that genetically diverse humans and mice share underlying mechanisms for these complex necrotizing responses, because humans with post-primary TB similarly develop thrombosis, coagulation necrosis, and lung infarction without organized granulomas [10, 11, 74]. Thus, the genetically diverse mouse models morphologically recapitulate all major forms of pulmonary TB in other species in a reproducible genetically controlled manner.

## Mechanisms of necrotizing responses as studied in mouse models

### Neutrophils as mediators of necrotizing responses to *M.tb*

Neutrophils are suicide bombers of the innate immunity with the primary function of killing extracellular pathogens. In this process of eliminating pathogens, neutrophils deploy numerous potentially host-damaging molecules such as matrix metalloproteases from cytoplasmic granules; abundant and potent oxidants; and the neutrophils themselves undergo necrosis releasing formyl peptides, complement C5a, and other molecules, which may stimulate “necrotaxis” (a type of neutrophil chemotaxis towards sites of cells and tissue death) [75–84]. These molecules from dead and dying bacterial and host cells are the most potent stimulators of neutrophil recruitment to sites of infection and cell/tissue death [79, 84]. In TB, large numbers of neutrophils accumulate in airways of pulmonary TB patients and in airways of mice that develop necrotizing responses [66, 72, 85–87] where dead neutrophils are packed in alveoli and terminal bronchioles within and adjacent to necrotizing granulomas [66, 72], presumably in response to macrophage death induced by rapidly replicating, virulent intracellular bacteria [40].

Neutrophils and their chemokines are markers of pulmonary TB in humans and in mice [40, 64, 66, 72, 73, 85–92]. Some evidence suggests that neutrophils contribute to TB because administration of antioxidants and anti-inflammatories (which may dampen neutrophil responses) decreased TB disease progression [93, 94] and because few neutrophils are present when bacillary replication is slowed [40]. However, only a handful of studies definitively link neutrophils [95] or their chemoattractants (e.g., CXCL5) [96] with increased susceptibility to *M.tb*, and others indicate that neutrophils are cellular biomarkers of failed immunity [92]. There are no studies that definitively prove that neutrophils drive necrotizing responses, and no studies have clearly defined genetic control of neutrophil recruitment. Definite hints exist because neutrophil responses vary with the hosts’ genetic architecture in humans and in experimentally infected mice [66, 85, 95, 97], and quantitative trait loci (QTLs) for TB susceptibility overlap with genes that may control neutrophil responses [91].

There are fundamental knowledge gaps in understanding how neutrophils contribute to macrophage and granuloma necrosis, but there are a number of candidate mechanisms to be tested. At the cellular (macrophage) level, neutrophils may cause lipid peroxidation of cell membranes, resulting in macrophage necrosis and release of bacilli. At the granuloma and lung tissue level, neutrophil influx could be more even more detrimental: (1) by causing lipid peroxidation of epithelial and endothelial cell membranes which irreparably damages lung tissue; (2) by proteolytic digestion of collagen-rich matrix and basement membranes of the lung alveolar septae; and (3) by physically obstructing airways which disrupt normal pulmonary function. We additionally theorize that neutrophils contribute to lung damage, which precedes cavitation by promoting thrombosis and infarction. This is supported by the facts that neutrophil granules contain pro-thrombotic molecules and that NETs from dying neutrophils also promote thrombosis [68, 98–100]. Additionally, this is supported by in situ observations of fibrin thrombi within capillaries adjacent to necrotic alveolar septae (Fig. 1d) as well as thrombosed vessels and necrotic lung tissue in patients with cavitary TB [11, 74].

Current evidence shows that neutrophil roles are complex. The abundance of neutrophil involvement in necrotizing responses and lesion progression does not prove, however, that neutrophil activity is the mechanistic cause of macrophage necrosis, granuloma necrosis, or lung tissue necrosis in TB lesions. At the early stage of infection, neutrophils can be protective by phagocytizing and killing a fraction of *M.tb* bacilli through oxidative mechanisms [101]. At the later stages, however, the excessive recruitment of neutrophils to TB lesions in the lung may serve as a common mechanism downstream of pathological reactions, which are triggered or maintained by other cell types. As an overall trend, in the susceptible mouse strains, neutrophils exacerbate susceptibility [95, 97]. However, it remains to be established whether this occurs because the susceptible mice have a genetic propensity for increased neutrophil recruitment to the lungs following *M.tb* infection, intrinsic functional defects of neutrophils, or it occurs downstream of other cells’ genetic defects.

In part, the paucity of known neutrophil-mediated mechanisms reflects scarce methodologies to manipulate and track neutrophils in vivo. Fortunately, new tools addressing roles for neutrophils have recently become available. This includes genetically diverse populations of mice (Diversity Outbred, Collaborative Cross) [102, 103]; immortalized neutrophil progenitor cells that can be manipulated in vitro and in vivo [104]; and neutrophil imaging technologies to track neutrophils in vivo over time in *M.tb-*infected mice. Eventually, those and other novel approaches will allow definitive mechanistic analyses of neutrophil contributions to the necrotization of TB lesions using mouse models, applicable to other species.

### Macrophage roles in necrotizing responses

A scrupulous work by the Kornfeld laboratory provided convincing experimental evidence that macrophage necrosis may trigger neutrophil recruitment to sites of *M.tb* infection [40, 105]. Using relatively resistant C57BL/6 mice infected with *M.tb* strains of varying virulence, they showed that higher neutrophil recruitment paralleled higher *M.tb* virulence and the rates of intracellular bacterial replication. Macrophage death in that model was driven by bacterial load (burst size), achieving which was followed by increase in uninfected and *M.tb-*infected neutrophils in the lungs [40]. This indicates that macrophage necrosis and release of *M.tb* indeed recruit neutrophils which phagocytize the extracellular *M.tb* bacilli. The authors also hypothesized that the neutrophil recruitment to sites of *M.tb* infection fundamentally reflects necrotaxis and that accumulations of dead cells and bacterial products may establish a self-sustaining positive feedback loop of neutrophil recruitment and death, even in the absence of bacterial replication. Thus, initially triggered by bacterial replication and macrophage death, neutrophil recruitment could accelerate lesion progression in autonomous manner and create the environment favorable for further *M.tb* replication. However, in the resistance B6 mice, this hypothetical amplification mechanism did not result in the formation of necrotic granulomas. Additional studies are necessary to determine whether targeting neutrophil recruitment can prevent or reverse the formation of necrotizing granulomas in those susceptible mouse strains, in which necrosis is caused by primary genetic defects of macrophages.

### Genetic studies of necrotizing granulomas

We addressed mechanism(s) of necrotizing granulomas using an unbiased forward genetic approach—“from phenotype to gene.” To map genetic loci controlling the formation of necrotizing granulomas in the lungs of C3HeB/FeJ (HeB) mice, we performed classical linkage analysis using crosses with the C57BL/6 (B6) mice [106, 107]. The B6 inbred strain is the most widely used inbred mouse strain in TB research, as a wild-type (wt) control for many genetically engineered (mostly knockout) mice of the same background. The wt B6 mice are permissive to infection with virulent *M.tb* but efficiently control *M.tb* replication due to the development of T cell-mediated immunity [57, 58]. Following infection, they typically survive greater than 12 months, and TB disease progression may reflect age-related changes in immune responses or immunological exhaustion [56] with up to 80 % of their lung occupied predominantly by macrophages and lymphocytes with interstitial fibrosis but lacking necrotizing granulomas.

Using the QTL analysis, we demonstrated a complex polygenic pattern of TB control, with both parental strains, B6 and HeB, carrying resistance and susceptibility alleles [108]. We focused our attention on four candidate loci (QTLs) at which the B6 mice carried resistance alleles on chromosomes 1, 7, 15, and 17. The chr.17 QTL overlapped with the mouse major histocompatibility complex (MHC) locus (H-2). Consistently, congenic mice that carried the C57BL/6-derived H-2^b^ allele on the C3H background developed more prominent Th1 response, both after *M.tb* infection and BCG vaccination. However, this effect was not sufficient to prevent the formation of necrotizing granulomas [109, 110]. To study the chromosome 1, 7, and 15 loci, we have generated a series of congenic mice by transferring large fragments of C57BL/6-derived chromosomes on the HeB background: each congenic strain received a fragment of one B6 donor chromosome. We found that only the B6-resistant allele at the chromosome 1 locus was necessary and sufficient to prevent the formation of necrotic granulomas after infection with virulent *M.tb* [61, 111]. The congenic HeB mice that carried the B6-derived allele at that locus lost their extreme susceptibility and resembled other standard substrains of C3H (C3H/HeJ, C3H/HeN, etc.) in terms of their survival and non-necrotic lung pathology [32, 112]. Therefore, we named the chromosome 1 locus *sst1*, for “super-susceptibility to tuberculosis.” When the *sst1* susceptibility allele was transferred from HeB to the resistant B6 background, the resultant congenic mouse strain B6.C3H-*sst1*^*S*^ also developed necrotizing granulomas in the lungs. Together, these results clearly demonstrated the dominant role of the *sst1* locus in controlling the formation of necrotizing granulomas [61].

Comparing mice that carried the *sst1*^S^ (susceptible) allele on two distinct genetic backgrounds, HeB and B6, we observed that the necrotizing granulomas developed only in the lungs, regardless of the route of *M.tb* infection—either the high dose intravenous (i.v.) or a low dose aerosol. The i.v. infections resulted in more rapid TB progression and death, as compared to the aerosol model. Nevertheless, there was a significant difference between the HeB and B6.C3H-*sst1*^S^ mice in median survival time (MST), 35 and 86 days, respectively, consistent with the overall higher TB resistance of the B6 mice due to the presence of resistance alleles at other, not linked to *sst1*, loci. However, the overall outcome was similar in both cases—mice died with large coalescing necrotizing lung lesions dominated by neutrophilic exudate and high mycobacterial loads, with the bacilli occupying intra- and extracellularly compartments [61, 107]. Those lesions closely resembled the type II lesions, according to classification of Irwin et al. discussed above [73].

A different type of lesion was observed in the lungs after a low dose aerosol infection (with a retained dose of *M.tb* 15–25 CFU at 24 h). Well-organized necrotizing granulomas developed in the lungs of both *sst1*^S^ mouse strains, HeB and B6.C3H-*sst1*^*S*^, 8–12 weeks post-infection. Remarkably, the development of necrotic lesions followed different trajectories and resulted in necrotic lesions different in their wall structure, as well as in the abundance and localization of the bacterial populations. At 5–6 weeks post-infection, the HeB developed coalescing tuberculous pneumonia. Abundant acid-fast bacilli were found within myeloid cells occupying alveolar spaces. Large necrotic foci could be found within the pneumonic areas at that stage surrounded by neutrophils that formed demarcation zone around necrotic foci. The appearance of organized necrotic lesions in the HeB mice followed the pneumonia and coincided with stabilized bacterial growth. In this model, the bacteria resided both extracellularly within the necrotic core, as well as inside macrophages within the granuloma wall, including the most outer layers and outside of the organized lesions.

**Fig. 2:**
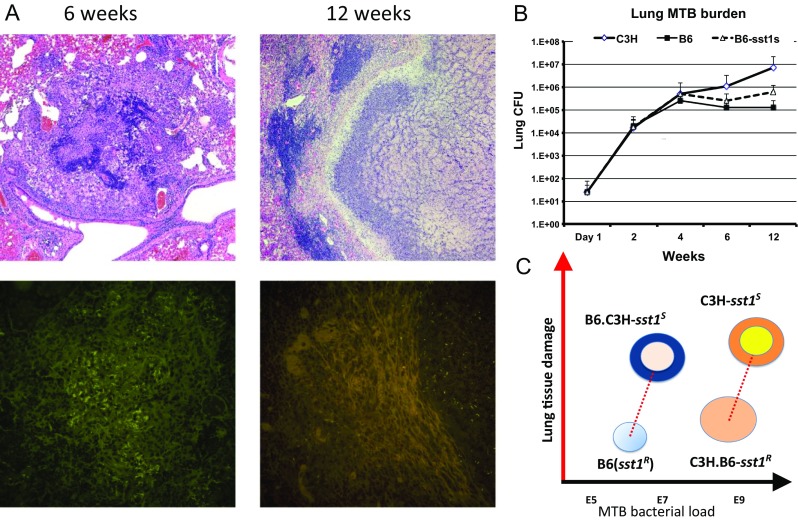
The *sst1* locus controls necrosis of TB granulomas. **a** TB granulomas in the lungs of B6.C3H-*sst1* mice 6 (*left panels*) and 12 weeks (*right panels*) after a low dose aerosol infection with virulent *M.tb* Erdman. TB granulomas double stained with hematoxylin and eosin (*upper panels*) and auramine-rhodamine (*lower panels*). The acid fast fluorescent staining with auramine-rhodamine identifies intracellular (at 6 weeks) and extracellular (at 12 weeks) *M.tb*. Caseous necrotic center containing *M.tb* is surrounded by organized wall at 12 weeks. **b** Total *M.tb* loads in the lungs of the parental C3HeB/FeJ (C3H) and C57BL/6J (B6) and the *sst1*-congenic (B6-*sst1S*) mouse strains. **c** Effects of the *sst1* locus and the host genetic background on lung tissue damage and *M.tb* lung burdens: the *sst1*-mediated control of granuloma necrosis (*Y*-axis) is uncoupled from the bacterial (*X*-axis)

Necrosis of TB lesions in B6.C3H-*sst1*^S^ followed a different pattern (Fig. 2). Initially, TB lesions in their lungs developed with kinetics similar to the B6 mice: after a period of rapid replication for 2 weeks, the bacterial loads were reduced five- to tenfold coinciding with activation of the adaptive immunity. By the sixth week, the bacterial load increased compared to B6 mice but remained 20–50 times lower than in the HeB mice (Fig. 2b). No alveolar exudates, pneumonia, necrotizing granulomas, extracellular bacteria, or neutrophil infiltration were observed at this stage. The lung lesions were compact, primarily composed of mononuclear phagocytes and lymphocytes (Fig. 2a). The extrapulmonary bacterial loads were low, demonstrating efficient systemic control of the bacterial replication. However, 9–12 weeks post-infection, large organized granulomas several millimeters in diameter, often occupying almost a whole lobe in the mouse lung, were formed. Compared to HeB, their wall was much thicker with more prominent fibrotic capsule adjacent to necrotic core. The characteristic concentric layers composed of macrophages, fibrosis, and tertiary lymphoid tissue formed around caseous center. The lymphoid layer contained CD4 and CD8-positive cells and CD-19-positive B cells organized in follicles. Few T cells penetrated the deeper layers of the lesion, while macrophages were predominant cells in contact with the necrotic masses. The bacteria were scarce compared to HeB and found almost entirely within necrotic core as single cells, suggestive of slow replication or non-replicative state. Occasionally, the intracellular bacteria were found within granuloma wall inside activated (iNOS-positive) macrophages but not outside the lesions. Obviously, the B6.C3H-*sst1*^S^ granulomas contained the bacterial spread more effectively compared to HeB. Consistent with more efficient control of *M. tb*, the B6.C3H-*sst1*^*S*^ mice survived significantly longer than the HeB mice—40 and 20 weeks after a low dose aerosol infection, respectively.

The above studies demonstrated complex genetic control of the necrotizing phenotype in the mouse model. First, it revealed the existence of a major genetic locus *sst1* specifically controlling the development of necrotizing granulomas per se, rather than necrosis being an outcome of a cumulative effect of many loci with equally important, but weaker, individual effects. Other genetic loci serve as quantitative modifiers: they determine specific immunological context and stages through which lesions reach the necrotic phase. Once necrotic foci appear, however, we observe some morphologic convergence on various genetic backgrounds. Perhaps necrotic products provide a morphogenic trigger for the lesion organization and pathogen containment via encapsulation. To sum, the genetic analysis of necrotizing granulomas in the HeB mouse model reveals a common underlying mechanism that can be genetically and functionally uncoupled from mechanisms of host resistance controlling the bacterial loads (Fig. 2c). The *sst1*-mediated mechanism is more suitably defined as a mechanism of cell or tissue resilience to inflammatory damage.

#### The *sst1* locus controls intrinsic macrophage function

The availability of the *sst1* congenic mouse strains allowed in-depth analysis of the cellular basis of the *sst1*-mediated phenotype. Using reciprocal bone marrow transplantation and adoptive transfer experiments, we established that *sst1* controlled innate immunity mediated by the bone marrow-derived non-lymphoid cells [9, 32, 113]. We also established that this mechanism controlled progression of infections caused by several taxonomically unrelated intracellular bacteria (*Listeria monocytogenes* [113], *Chlamydia pneumoniae* [114], and *Francisella tularensis* LVS (in preparation). A candidate gene *i*ntracellular *p*athogen *r*esistance 1 (*Ipr1*) encoded within *sst1* locus and expressed in activated macrophages has been identified using positional cloning [32]. We reported that mouse macrophages isolated from *Ipr1*-deficient mice and infected with intracellular pathogenic bacteria in vitro die more readily via non-apoptotic, presumably necrotic, death. Recent work on characterization of the *sst1*/*Ipr1*-mediated pathway in macrophages is beyond the scope of this review and will be published elsewhere (Bhattacharya et al., in preparation). Suffice it to say that *Ipr1* is a nuclear protein induced in activated macrophages and involved in control of macrophage stress responses. Its loss of function makes the *sst1* susceptible macrophages, HeB and B6.C3H-*sst1*^S^, more sensitive to stress and prone to death upon infection with intracellular bacteria.

This mechanistic insight provides an explanation for incomplete protection offered by BCG vaccination in HeB mice. On the one hand, the BCG vaccine induces protective acquired immune response in HeB mice that reduces *M.tb* growth and extends their survival [9, 110, 115, 116]. Obviously, earlier activation of mycobacteria-specific Th1 response in BCG vaccinated animals produces a significant modifier effect on the *sst1*S phenotype. However, it does not remedy the mechanistic root cause of necrosis in macrophages and the necrotic lesions still develop, albeit with significant delay in time [9]. Therefore, the *sst1* deficiency of macrophages poses a limit to BCG vaccination efficiency. Because current vaccine candidates against *M.tb* aim at activating macrophages via T cell-mediated help, even highly immunogenic vaccines may fail in immune competent hosts whose TB susceptibility is based on certain macrophage defects.

Together, our findings on a specific role of the *sst1* pathway in macrophages extend the burst size hypothesis of Kornfeld et al., which has been established in B6 mice. We suggest that the burst size being intrinsic to macrophages is not a fixed property but may vary depending on host genetic background and environment, which determine macrophage resilience. Meanwhile, the effector mechanisms of resistance control the rates of intracellular bacterial survival and replication, i.e., determine whether and how fast a certain burst size is achieved. Therefore, interactions of resistance and resilience mechanisms would produce cumulative effects that determine the lesion dynamics and types of immunopathology. We anticipate that this framework is broadly applicable to the analysis of the complex genetic control of host interactions with *M.tb* and other intracellular pathogens [117].

A work on dissecting the genetic control is ongoing in other mouse models of necrotic TB inflammation. Using a cross of I/St and B6 mice, Apt and colleagues have mapped three QTLs on chromosomes 3, 9, and 17 [118–120]. The chromosome 17 QTL has been narrowed down to a smaller candidate region encompassing mouse MHC using a series of interval-specific congenic mice. Functional analysis demonstrated that the susceptible I/St-derived MHC allele was associated with higher bacterial loads, more pronounced inflammation, and lower Th1 cell response in the lungs after aerosol infection with virulent *M.tb* [121–123]. In yet another model of necrotic TB inflammation in DBA/2 mice, two QTLs have been mapped to chromosomes 8 and 19 also in a cross with C57BL/6. Of those, the chromosome 19 QTL (named *trl4*) specifically controlled the bacterial replication in the lungs after aerosol infection with *M.tb* [124, 125]. It remains to be established in those models whether lung necrosis is epistatically controlled by a major locus in a manner similar to *sst1*, or the necrotic phenotype is due to additive effects of several susceptibility loci controlling the bacterial replication.

A more comprehensive dissection of pathways to necrotizing granulomas may be achieved using genetically heterogeneous populations, such as Diversity Outbred mice, which are as diverse as the human population. A substantial benefit of this particular mouse model is that Diversity Outbred genomes are suited for high-resolution genetic mapping and have already identified novel genetic associations with a variety of traits [102, 103, 126–129], providing proof of concept the same will be true for TB research. The genetic heterogeneity comes from eight distinctive founder strains of which five are inbred laboratory strains and three are wild-derived strains [102, 129]. Only three (C57BL/6, 129, and A/J) have been used in *M.tb* research, identifying the importance of Th1-mediated resistance and providing some insight into susceptibility [56, 130–132]. Responses of the other five founder strains (NOD/LtJ, NZO/HlLtJ, CAST/EiJ, PWK/PhJ, and WSB/EiJ) have not yet been published.

DO mice have been used in only three *M.tb* studies one of which included vaccination responses only, not infection [65, 66, 85]. Following aerosol infection, approximately half of DO mice developed necrotizing lung granulomas with neutrophilic tuberculosis pneumonia which resembled type II lesions described in [73] (Fig. 1a–d). All parental mouse strains are immune competent. The fact that excessive neutrophil recruitment and abundant neutrophil chemokines were common TB disease correlates in Diversity Outbred mice [66] suggests that necrotizing granulomas and neutrophilic tuberculosis pneumonia may be triggered by multiple genetic loci whose interactions control lung damage caused by TB infection. Some of those loci may work upstream of neutrophil recruitment controlling macrophage death. In this situation, neutrophils may serve as bystanders but it is, more likely, that neutrophils amplify tissue damage initiated by other causes. Meanwhile, other genetic loci may increase cell-autonomous neutrophil activity [133, 134]. Both possibilities would be consistent with the ameliorating effect by neutrophil blockade, as demonstrated in several studies where positive effects of neutrophil depletion were detected but only in strains already prone to developing necrotizing granulomas and not in resistant B6 mice [94, 95, 97, 135].

We anticipate that high-resolution genetic mapping followed by identifying stages of the disease progression, cell types, and functions controlled by individual genetic loci in diverse mouse models may reveal distinct pathways for necrotizing granuloma formation and progression, as well as mechanistic points of their convergence.

## TB spectrum: of mice and men

The fundamental difference between the human and mouse species as hosts for *M.tb* is that modern human populations are a product of long co-evolution with *M.tb*, while mice are not. In human populations exposed to *M.tb*, the highly susceptible individuals are selected against and genetic variants that confer high degree of susceptibility to TB remain at low frequency. Also, there is a significant proportion of innately resistant individuals that remain infection-free even in high exposure settings, ranging between 20 and 70 % in different studies. Mice are not naturally infected with *M.tb*, do not transmit the bacteria via aerosols, and were not subjected to natural selection by this pathogen. Not surprisingly, the human and mouse TB spectra do not fully overlap. As currently known, the spectrum of natural genetic variation in TB susceptibility among laboratory mice is shifted towards susceptibility as compared to modern human populations. Below, we propose operational stratification of humans in terms of TB susceptibility and identify matching genetically defined mouse strains (Table 1).

**Table 1:** TB spectrum and corresponding genetic mouse models

Strata	*M.tb* control	Clinical forms	Genetic control vs environment	Immune mechanisms		Mouse model	Necrosis in TB lesions
				Innate	Adaptive		
IR	Remain uninfected after repeated exposure to *M.tb*	N/A	Genes and environmental factors are unknown	Sufficient to eradicate *M.tb*	Importance unknown, as Th1 activation is not detectable	Currently not available	N/A
Innately							
Resistant							
PR	Permit initial replication of *M.tb* but control the disease progression	Latent TB infection (LTBI) Reactivation TB	Genes that differentiate from IR are unknown Environmental factors play dominant roles in reactivation	Subtle defects: not sufficient to eradicate *M.tb*	Th1 immunity is adequate for control TB progression	B6	No
Permissive							
Resistance							
PS	Permit initial *M.tb* replication and progression towards the disease	Primary progressive TB, disseminated and pulmonary TB	Complex polygenic control Environmental factors are important	Not sufficient to eradicate *M.tb* and to control TB progression	Th1 immunity is not sufficient to control TB progression; no apparent immune deficiency	BALB/c C3HeB/FeJ B6-*sst1* ^*S*^ CBA I/St DBA/2 A/J	No Yes-NP, NG Yes-NG Yes-NG Yes-NG Yes-NP Yes-NP
Permissive							
Susceptible							
ES	Permit replication of environmental and avirulent mycobacteria; disseminated infection due to inactivation of an essential mechanism	Mendelian susceptibility to mycobacterial diseases (MSMD)	Monogenic control: Genetic and environmental modifiers may exist, are unknown	Not sufficient to eradicate avirulent mycobacteria	Th1 immunity is severely compromised due to either defective IFNγ production or unresponsiveness to IFNγ	B6-*ifng* ^*tm*^ B6-*stat1* ^*tm*^ B6-*irf1* ^*tm*^ B6-*prdkc* ^*tm*^	Yes-NP
Extremely							
Susceptible							

*NP* necrotizing TB pneumonia, *NG* necrotizing TB granuloma

In the order of decreasing resistance, humans can be classified as innately resistant (IR), permissive resistant (PR), permissive susceptible (PS), and extremely susceptible (ES).

The IR individuals remain infection free after repeated exposure to *M.tb*. presumably due to effector mechanisms of innate immunity that effectively eliminate *M.tb* before it establishes the primary infection site in the lung. Those mechanisms might be evolutionary acquired and refined only in humans. Macrophage-mediated killing of mycobacteria via vitamin D-stimulated production of bactericidal peptides is one of them. Perhaps other mechanisms also exist, but their discovery is complicated, exactly because of a lack of appropriate animal models. To date, no IR equivalent has been identified among standard laboratory mouse strains. Upon experimental infection, each mouse strain is permissive to some degree and remains either chronically infected (PR equivalent) or develop primary progressive TB (PS equivalent). The genetic analysis of human cohorts that remain infection free in high exposure settings, including HIV-infected patients, is perhaps the best approach to discovering human-specific IR effector mechanisms. Subsequently, mouse models can be genetically engineered to reproduce those mechanisms using various methods to “humanize” mouse genes.

The ES individuals represent another phenotypic extreme in humans—monogenically controlled severe mycobacterial diseases (MSMD). Inactivating mutations in essential pathways of antituberculosis immunity in MSMD patients results in susceptibility to normally apathogenic environmental mycobacteria and avirulent vaccine strain of *M. bovis* BCG [136, 137]. In those individuals, disseminated mycobacterial diseases develop due to unrestricted replication of mycobacteria, and necrosis within lesions is driven by high bacterial loads. Albeit rare, those conditions represent a significant therapeutic challenge. In this category, the mouse and human studies demonstrated remarkable complementarity. First, the devastating effects of inactivating mutations in genes of IL12-IFNγ axis and IRF8 on resistance to *M.tb* were described in mice [138–140]. These experimental findings correctly predicted extreme susceptibility to mycobacteria among the human mutation carriers. Each mutation inactivating the essential pathway of host resistant to *M.tb* produces strong independent effect irrespective of genetic modifiers, explaining the monogenic, Mendelian, pattern of inheritance. The discovery of novel MSMD genes using exome re-sequencing in affected humans [141] can be mechanistically followed-up using genetically engineered mice with various types of mutations introduced in a corresponding gene. The mutants, either natural or genetically engineered, would serve as accurate models of MSMDs for both mechanistic studies and therapeutic applications.

The majority of human tuberculosis spectrum rests between the above extremes and is represented by groups of people who are permissive for tuberculosis infection but less susceptible than the ES group. Those individuals can be further subdivided into PR and PS subpopulations. The PR population allows implantation of *M.tb* and establishing chronic asymptomatic infection, while the PS population allows progression of the infection towards overt disease within months of the initial exposure.

Most of the mouse strains that develop necrotic lung lesions match the PS category within the human spectrum. Thus far, the mouse model of necrotic TB lesions in C3HeB/FeJ (HeB) has been evaluated and successfully adopted by several research groups. Pathomorphological diversity of TB lesions in HeB mice and their similarity to necrotic lesions in other animal models and humans enabled modeling physiologically relevant states of the bacteria and allowed the analysis of drug distribution within necrotic lesions [62, 72, 73, 142–144]. Other mouse strains that develop necrotic TB lesions, such as CBA, I/St, A/J, and DBA/2, may be used to expand the lesion diversity, which is desirable for preclinical testing of antibacterial drugs.

From the mechanistic standpoint, perhaps no single inbred mouse strain is sufficient to reproduce the heterogeneity of pathways leading to the necrotic phenotypes. All of the above mouse strains possess essential mechanisms of host resistance to TB, but the genetic basis underlying their susceptibility most likely differ between the strains. As discussed above, those mouse phenotypes represent complex genetic traits, where allele combinations shape the phenotypes by producing quantitative and threshold effects. Their discovery via systematic forward genetic analysis should reveal genes and pathways that play important roles in controlling progression toward necrotic TB lesions in mice. Corresponding mutations may not be sufficiently represented in modern human populations, because of their elimination via negative selection by TB. Due to their low frequency, they may remain undetectable in human genome-wide association studies (GWAS). Nevertheless, those pathways may be compromised in humans, as well, due to environmental factors, such as co-infections, co-morbidities, and stress among others. Therefore, mechanisms of susceptibility discovered in mice may serve as valid targets for host-directed therapies (HDTs) in humans, while a corresponding mutant mouse strain would serve as a genetically defined model for preclinical evaluation of HDTs targeting a specific pathway.

The PR, human hosts control the pathogen, survive through reproductive age, and are not subjected to intense negative selection [145]. The PR individuals do not readily eradicate *M.tb*; however, their immunity is adequate for the disease prevention and remains so during lifetime in the majority of LTBI cases. We believe that this group produces the biggest epidemiological impact for two reasons: first, it represents a large reservoir of latent *M.tb* infection (LTBI) and, second, yields individuals that may efficiently transmit the bacteria via aerosols upon the LTBI reactivation. Therefore, dissecting mechanisms of TB control in this group using animal models is especially important.

Perhaps, the best-studied mouse model resembling PR humans is represented by the commonly used C57BL/6 (B6) mice. These mice control infection with *M.tb* delivered either by aerosol or i.v. routes. After a low dose aerosol infection, a period of rapid *M.tb* replication lasts for approximately 2 weeks during which *M.tb* doubling time roughly approaches the doubling time in liquid culture, i.e., the bacterial growth proceeds in unrestricted manner. After reaching the peak in the lungs, the bacterial load is reduced five- to tenfold commensurate with the onset of T cell response in regional lymph nodes and migration of *M.tb*-specific T cells into the lung lesions. Those stable levels are maintained for several months to a year, and no necrotic granulomas have been observed in un-manipulated animals, indicating that pathways that restrict necrosis in TB lesions remain intact. C57BL/6 mice eventually die as the lungs become filled with degenerating macrophages and lymphocytes and development of concurrent septal fibrosis. The same type of lesions in humans could correspond to latent infection. The BCG vaccination of B6 mice prior to infection further reduces the bacterial loads, but neither prevents the infection nor results in *M.tb* eradication.

Pathways responsible for necrosis in TB granulomas can be identified using genetic analysis in crosses of mice representing the PR-like (B6) and the PS-like (I/St, CBA, A/J, and DBA/2) mouse strains as well as the DO and Collaborative Cross mice. Genetic mutations identified in the PS-like mice would point towards pathways, whose inactivation result in granuloma necrosis. We suggest that in PR humans, those pathways may be initially intact but become compromised over time due to non-genetic, environmental, causes, creating phenocopies of mouse mutations. For example, mice that carry the *sst1* susceptible allele on the B6 background may recapitulate phenotypes associated with functional inactivation of the human homologue of Ipr1, SP110. The inactivating mutations in Sp110 gene are present in human populations at a very low frequency, because they cause severe immune deficiency and susceptibility to opportunistic infection *Pneumocystis jirovecii* and CMV [146]. However, a number of studies found that the SP110 protein directly interacts with viral proteins and is involved in control of macrophage activation and death caused by chronic viral infections, such as EBV and hepatitis C virus [147, 148]. We hypothesize that in human macrophages latently infected with certain viruses, the expression of viral proteins may be upregulated within inflammatory lesions, such as granulomas. Binding and sequestering SP110 by viral proteins would neutralize its activity and create a phenocopy of the SP110 deficiency in macrophages within granulomas, while systemic immunity remains intact. This hypothetical scenario emerges from the analysis of the Sp110 loss of function at the whole organism level in mice and biochemical characterization of viral protein interactors in isolated human cells in vitro. It suggests a novel mechanism of the necrotic granuloma development in immunocompetent humans, whereby viral co-infections that primarily affect activated macrophages may play a leading role. This example demonstrates that the genetic dissection of TB in mouse models is capable of identifying pathways and providing testable hypotheses relevant to TB pathogenesis in real-life human populations.

To conclude, the fact that laboratory mice do not copy all aspects of human disease does not invalidate the mouse model. On the contrary, if researchers stratify human phenotypes and use genetically defined mouse models to match them, the human and mouse studies demonstrate remarkable complementarity in the analysis of TB pathogenesis at the cellular and whole organism levels.

## Future directions

Because traditional mouse models and basic readouts were not sufficiently aligned with other animal models and human TB, they deemed unreliable for mechanistic and preclinical studies. These disputes during past decade have led to a situation where tremendous progress in mouse genetics, genetic engineering, and imaging has not been satisfactorily incorporated into translational TB research. The situation started to change with the broader acceptance of the C3HeB/FeJ model of necrotic TB lesions for preclinical drug testing and in vivo imaging [62, 142]. However, as discussed above, no single “mouse TB” model can reflect the mechanistically and morphologically diverse forms of human TB. Therefore, the question should not be “whether or which mouse is a good model for human TB.” Instead of comparing abstract “human and mouse TB,” more relevant questions are how to best use the existing and how to create new mouse models that resemble specific forms of the human disease more accurately.

A particularly important application of the advanced mouse models, uncontested by other methods, would be to study the dynamics of human-like TB lesions in the lungs. In mice, as in humans, advanced TB primarily targets and damages the lung. Currently, the mechanistic basis of this key evolutionary trait remains largely unknown. So far, most of the data on cell interactions in granulomas was obtained using high-resolution live imaging in zebrafish model and extrapolated to human TB. These pioneering “proof of principle” imaging and genetic studies now can be extended to study specific contributions of the lung environment at different stages of the granuloma progression and regression.

To date, many fundamental questions remain unanswered with regard to pulmonary TB. For example, no experimental data exist on the rate of macrophage survival in TB granulomas. What is the time between a monocyte recruitment and death in granulomas? Does the balance of cell death, clearance of dead cells, and monocyte recruitment determine the appearance and the dynamics of necrotic lesions? How the macrophage death modality affects the granuloma dynamics in vivo? How much replication of immune cells occurs locally in granulomas? Do antigen-specific T cells directly interact with infected phagocytes in different granuloma layers? What is the outcome of these interactions? What are specific roles of T lymphocytes in necrotization of TB granulomas? Do T cells induce or prevent the necrotization? Does the neutrophil recruitment to granulomas precede or does it follow the formation of micro-necrotic lesions? Ultimately, answering these questions must reveal why effective systemic immunity is not sufficient to control *M.tb* in lung granulomas and how necrosis develops. Determining which factors can shift the granuloma dynamics towards or away from the necrotic trajectory is crucial for developing rational granuloma-directed therapies to prevent or reverse the necrotization process. We suggest that such anti-necrosis therapies may be directed at (i) T cells—to activate or suppress specific subsets; (ii) macrophages—to increase macrophage longevity and resilience or prime them for activation; and (iii) neutrophils—to block their recruitment or enzymatic activities.

New quantitative criteria and tools for the analyses of TB granuloma progression in mice have to be developed for in-depth mechanistic analyses that also translate well into preclinical studies. For example, using novel genetic and genetic engineering tools, new generation mouse models can be created that not only recapitulate a particular trait of the human disease but also carry imaging and lineage tracking reporters for its rapid quantitative analysis. In vivo and ex vivo imaging techniques, such as MicroCT, PET, SPECT, and optical imaging with an expanding array of functional imaging probes provide a holistic view of pulmonary TB and allow quantitative spatio-temporal assessment of individual TB lesions based on inflammatory markers, myeloid cell recruitment, and necrosis [144, 149]. Additionally, computer algorithms are now being developed for automated detection of the cellular composition in TB lung lesions at microscopic level [150] and eventual quantification and reconstruction/modeling. Applying multi-modal imaging approaches will facilitate functional analyses of host-pathogen interactions in situ—within dynamic granulomas in lung-specific context. Combination of the new generation mouse models with advanced imaging technologies will also allow rapid and inexpensive assessment of experimental vaccines and therapies prior to testing in larger animals and clinical trials.

Bringing the new generation mouse models and readouts to the field will also bolster the analyses of *M.tb* virulence and pathogenesis in vivo*. M.tb* mutants and clinical isolates can be systematically assessed for their ability to induce, and survive within, lung necrotic granulomas. Mechanisms of *M.tb* reactivation from latency and translocation from necrotic core outside granulomas, acquisition of drug tolerance, and evolution of drug resistance, as well as drug distribution [143], all can be studied in a context of mouse necrotic lung lesions in combination with bacterial reporters and advanced tools for inducible gene expression and silencing.

In conclusion, the role that mouse models play in basic and translational TB research will continue to increase allowing researchers to address fundamental questions of TB pathogenesis and bacterial physiology in vivo with unparalleled depth. This knowledge is not merely a pursuit of academicians but a rational search to identify molecular targets that control necrosis, a key evolutionary determinant of *M.tb* propagation in humans, to develop effective necrosis-directed therapies and to test them in vivo with efficiency, mechanistic depth, and quantitative rigor currently attainable only in a mouse model.
